# Pharmacokinetics, distribution, metabolism, excretion and safety characterization of ZJCK-6-72: novel DYRK1A inhibitor with optimized brain exposure for Alzheimer’s disease therapy

**DOI:** 10.3389/fphar.2026.1792258

**Published:** 2026-05-01

**Authors:** Zhenshu Li, Huanhua Chen, Xinzhu Li, Aizhu Yang, Xinpeng Wang, Siyuan Liu, Xinyue Ning, Lijun Zhou, Fangyuan Zheng, Fanghua Xun, Bei Hu, Yaling Cui, Dong Yao, Xudong Gao, Zihua Xu, Qingchun Zhao

**Affiliations:** 1 Department of Clinical Pharmacy, General Hospital of Northern Theater Command, Shenyang, China; 2 Graduate School of China Medical University, China Medical University, Shenyang, China

**Keywords:** ADMET, DYRK1A, metabolism, pharmacokinetics, unbound brain exposure, ZJCK-6-72

## Abstract

Developing effective drugs for Alzheimer’s Disease (AD) is critically limited by insufficient brain exposure. To address this, structural optimization was employed to balance physicochemical properties and enhance blood-brain barrier (BBB) permeability, resulting in the development of ZJCK-6-72, a novel DYRK1A inhibitor. This study aims to comprehensively evaluate the drug-like properties and safety profile of ZJCK-6-72.

**Methods:**

A comprehensive *in vitro* and *in vivo* ADMET (absorption, distribution, metabolism, excretion, and toxicity) profiling of ZJCK-6-72 was conducted. Key assessments included pharmacokinetics, tissue distribution studies in rats, plasma protein binding assays, and metabolic stability testing. Additionally, acute toxicity was evaluated to determine the safety margin, and enzyme inhibition assays were performed to assess potential drug-drug interactions.

**Results:**

ZJCK-6-72 exhibited excellent oral bioavailability (78.03%) and extensive tissue distribution in rats. Notably, the compound demonstrated substantially enhanced brain penetration, with a brain-to-plasma ratio ranging from 1.92 to 4.63. The unbound brain-to-plasma partition coefficient (K_p, uu_) was determined to be 3.50, significantly exceeding unity. Metabolic studies indicated a shift towards CYP1A2 and CYP2C19 mediated pathways, with concentration-dependent inhibition observed (IC_50_ ≈ 1.8 μM). The acute toxicity assessment revealed an LD_50_ of 233.9 mg/kg bodyweight.

**Discussion:**

The high K_p, uu_ value indicates that the efficient brain entry of ZJCK-6-72 is likely driven by active uptake mechanisms rather than simple passive diffusion, confirming its ability to effectively evade efflux transporters. Although the inhibition of CYP1A2 and CYP2C19 suggests a potential for mechanism-based auto-inhibition, the acute toxicity profile demonstrates an acceptable safety margin for therapeutic development.

**Conclusion:**

ZJCK-6-72 displays an optimized pharmacokinetics profile characterized by superior unbound brain exposure. These findings support the potential of ZJCK-6-72 as a promising lead candidate for CNS-targeted AD therapy.

## 1 Introduction

Alzheimer’s disease (AD) is a neurodegenerative disease characterized by progressive cognitive decline and memory loss, with key pathological features comprising amyloid β accumulation and hyperphosphorylated tau protein (Singh et al., 2024). The disease poses a substantial and growing burden, currently affecting 6.9 million older Americans and exhibiting a more than 140% rise in mortality since 2000, establishing it as a major cause of death (Alzheimer’s Disease Facts and Figures, 2024). Increasing AD prevalence presents considerable global challenges for patients, accompanied by significant social and economic consequences. Existing pharmacotherapies such as the recently FDA-approved lecanemab deliver only symptomatic relief with restricted therapeutic benefits (Singh et al., 2024; Jönsson et al., 2023; Passeri et al., 2022; Yadollahikhales et al., 2023), underscoring the pressing demand for novel therapeutic targets.

Selective inhibitors constitute a promising drug class aimed at specific enzyme or pathways involved in AD pathology to reduce off-target effects and improve treatment safety. The dual-specificity tyrosine(Y)-phosphorylation-regulated kinase 1A (DYRK1A) gene resides in the Down syndrome critical region of human chromosome 21 and encodes the beta-amyloid (Aβ) precursor protein (APP). Elevated DYRK1A expression contributes significantly to Alzheimer’s disease progression through its phosphorylation of amyloid precursor protein and subsequent promotion of Aβ peptide formation (Fernandez Bessone et al., 2022; Souchet et al., 2019). Branca et al. reported that sustained pharmacological inhibition of DYRK1A activity effectively attenuates both amyloid deposition and tau hyperphosphorylation while rescuing cognitive impairments in 3×Tg-AD mice (Branca et al., 2017). The dysregulation of DYRK1A is also correlated with other diseases like Down syndrome, Parkinson’s disease, diabetes, and cancer. This multifunctional involvement in diverse disease pathways has established DYRK1A as a prominent focus in current neuropharmacological research.

Recent years have witnessed considerable expansion in DYRK1A inhibitor development, with numerous compounds emerging as candidate therapeutics for neurological disorders (Liu et al., 2022; Zhang et al., 2024). However, many promising inhibitors face pharmacological challenges, including suboptimal pharmacokinetic profiles, incomplete safety characterization, and critically insufficient BBB penetration. The DANDY chemical series exemplified this limitation, where pronounced *in vitro* potency and selectivity were offset by structural features predisposing to extensive glucuronidation and poor brain exposure (Gourdain et al., 2013). Similarly, the natural alkaloid harmine demonstrates strong DYRK1A inhibition but suffers from limited oral absorption and non-selectivity through monoamine oxidase A interaction, creating safety complications (Äbel et al., 2025; Göckler et al., 2009). Our earlier compound ZJCK-6-46 addressed certain limitations with nanomolar enzymatic inhibition and adequate systemic exposure, yet it exhibited insufficient brain distribution, limiting its therapeutic potential for central nervous system targets (Chen et al., 2024). These examples highlight how inadequate drug-like properties can obstruct the development of otherwise potent DYRK1A inhibitors, emphasizing the continued need for optimized candidates with balanced efficacy and pharmacokinetic characteristics for advancing Alzheimer’s disease treatment.

Based on the critical target DYRK1A and employing a pharmacophore fusion-guided molecular design strategy, we developed ZJCK-6-72 as an optimized candidate. To address the limitation of poor CNS distribution observed in ZJCK-6-46, our subsequent molecular optimization focused on balancing lipophilicity and metabolic stability to enhance intrinsic blood-brain barrier (BBB) penetration. The enzymatic inhibitory activity against DYRK1A was assessed prior to this ADMET study, revealing that ZJCK-6-72 maintained nanomolar potency (IC_50_ = 8.64 nM) relative to the precursor ZJCK-6-46 (IC_50_ = 0.68 nM). We hypothesized that the structural refinements in ZJCK-6-72 would translate into a superior pharmacokinetic profile, particularly increasing the unbound drug concentration in the brain, which is the driving force for pharmacological activity.

To test this hypothesis and unravel the underlying pharmacokinetic and metabolic mechanisms enabling its improved CNS exposure, we conducted a comprehensive ADMET investigation. This study aims to quantitatively characterize the pharmacokinetics, tissue distribution, and excretion of ZJCK-6-72 in rats, identify the primary metabolic enzymes and pathways, assess its drug-drug interaction potential, and evaluate its preliminary safety profile, with direct comparison to ZJCK-6-46.

## 2 Materials and methods

### 2.1 Chemicals and reagents

**FIGURE 1:**
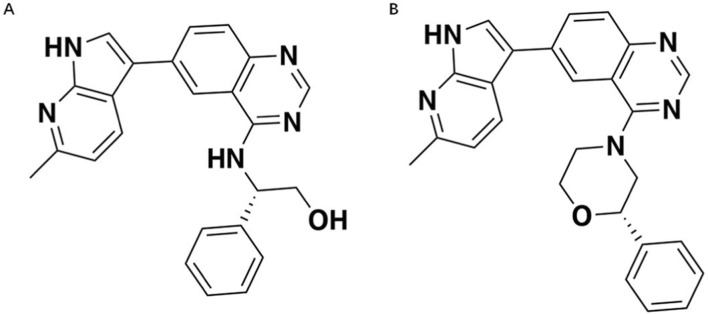
The chemical structure of **(A)**. ZJCK-6-46; and **(B)**. ZJCK-6-72.

The compound ZJCK-6-72 (chemical purity: ≥98%) was synthesized in our laboratory. The DYRK1A enzyme inhibition IC_50_ value for ZJCK-6-72 was obtained from a separate, unpublished study conducted by our group. Carbamazepine (purity ≥98%, wkq23052503), employed as the internal standard (IS), was sourced from Sichuan Weikeqi Biological Technology Co., Ltd. The molecular structures of ZJCK-6-46 and ZJCK-6-72 are depicted in Figures 1A,B, respectively. HPLC-grade methanol and acetonitrile were obtained from Merck (Germany). Deionized water was acquired from Wastons (China), and LC-MS grade formic acid for mobile phase preparation was procured from Macklin (China). All metabolic incubation studies utilized the Phase I Metabolic Stability Kit (Sprague-Dawley rat liver microsomes and human liver microsomes, mixed gender) and the CYP450 Phenotype Characterization Kit (with 7 selective inhibitors, human liver microsomes, mixed gender), both supplied by IPHASE Biosciences (China).

### 2.2 Animals

All animal experiments received approval from the Animal Ethics Committee of the General Hospital of Northern Theater Command (Approval No.: 2025-025). Male Sprague-Dawley rats and C57BL6/J mice, originally supplied by Changsheng (Liaoning, China) Biotechnology Co., Ltd., were bred and maintained within our institution’s specific pathogen-free animal facility. The study was conducted in compliance with the institutional guidelines for the ethical use and care of laboratory animals.

### 2.3 *In vivo* pharmacokinetic, distribution, and excretion studies

#### 2.3.1 Sample preparation

Biological samples were processed using matrix-specific protocols. Plasma aliquots (50 μL) were combined with 10 μL of internal standard working solution and protein-precipitated with 200 μL of acetonitrile. The mixtures were thoroughly vortexed for 3 min and centrifuged at 16,000 g for 10 min at 4 °C, with resulting supernatants directly injected for analysis.

Tissue specimens (approximately 300 mg) underwent homogenization in 900 μL of acetonitrile-saline solution (1:1, v/v). Following centrifugation under identical conditions, 50 μL of supernatant was subjected to the same processing protocol as plasma samples.

Urine samples were prepared according to the plasma procedure. Fecal samples were desiccated for 24 h prior to processing via the tissue sample protocol. Specimens exceeding the calibration range were appropriately diluted with blank matrix to maintain quantitative accuracy.

#### 2.3.2 HPLC-MS/MS method

Quantitation of ZJCK-6-72 in biological matrices was achieved using a validated HPLC-MS/MS system. Chromatographic separation employed a WATERS XTERRA MS C18 column (100 × 2.1 mm, 3.5 μm) maintained at 40 °C. The mobile phase consisted of 0.1% aqueous formic acid (A) and acetonitrile (B) delivered at 0.3 mL/min with gradient elution: 90% A (0–1.0 min), linear transition to 10% A (1.0–3.0 min), isocratic elution (3.0–3.5 min), return to initial conditions (3.5–3.7 min), and re-equilibration until 5.1 min.

Mass spectrometric detection was performed using an AB Sciex API3200MD instrument with electrospray ionization in positive mode. Multiple reaction monitoring transitions were m/z 422.2→302.3 and 422.2→275.3 for ZJCK-6-72, and m/z 237.2→194.2 for carbamazepine (IS). Optimized parameters included: ion source temperature 500 °C, spray voltage 5.0 kV, collision gas pressure 5 psi, and nebulizer/heater gas flows at 50 psi each.

The bioanalytical method demonstrated satisfactory specificity, sensitivity, linearity, accuracy, precision, and stability according to current regulatory standards, meeting all validation criteria for quantitative determination in biological matrices, with detailed validation data and figures provided in Supplementary Section 1.

#### 2.3.3 Pharmacokinetic and bioavailability study of ZJCK-6-72 in SD rats

Before the initiation of the experiment, SD rats (weighing 180–200 g) were acclimatized for 14 days under controlled environmental conditions with a temperature of 25 °C and a relative humidity of about 40%–70%. The animals were fasted overnight (at least 8h) before administration, with water available and libitum.

ZJCK-6-72 was dissolved in a mixed solvent consisting of 10% DMSO, 5% Tween-80, 40% PEG-400, and 45% saline. The rats were randomly assigned to two groups (n = 6 per group), and received a single dose of ZJCK-6-72 either intravenously (5 mg/kg bodyweight, 2 mL/kg) or orally (20 mg/kg bodyweight, 5 mL/kg).

The doses for the bioavailability study were selected based on methodological feasibility: 5 mg/kg bodyweight for intravenous administration was chosen to ensure formulation tolerability and animal safety, while 20 mg/kg bodyweight for oral administration was used to maintain plasma concentrations above the quantification limit for accurate PK parameter estimation.

Blood samples (0.3–0.5 mL) were obtained from the jugular vein into EDTA-K2-treated tubes at predetermined intervals including pre-dose, 0.25, 0.5, 1, 2, 3, 4, 6, 8, 12, and 24 h following compound administration. Plasma separation was achieved by centrifugation at 3,500 rpm for 10 min, with all samples maintained at −80 °C pending subsequent analysis. Sample processing involved protein precipitation with acetonitrile, and the resulting supernatants were subjected to quantitative analysis using a validated HPLC-MS/MS methodology. The absolute oral bioavailability (F_ab_) was determined by comparing the area under the concentration time curve (AUC) values following oral and intravenous administration according to the standard pharmacokinetic equation.

$$F_{ab}=\frac{{AUC}_{po}\;*\;{Dose}_{iv}}{{AUC}_{iv}\;*\;{Dose}_{po}}\;*\;100\%$$

#### 2.3.4 Distribution study of ZJCK-6-72 in SD rats

Forty male SD rats (body weight 180–220 g) received a single 20 mg/kg bodyweight oral dose of compound ZJCK-6-72, which is the same dose used for the oral pharmacokinetic and excretion studies to maintain experimental consistency. To characterize the compound’s distribution kinetics, subgroups of five animals were sacrificed under isoflurane anesthesia at designated intervals (1, 2, 4, 6, and 8 h post-administration). Immediately following euthanasia, systemic vascular perfusion with chilled saline was performed through cardiac puncture to eliminate residual blood components from the circulatory system.

Major organs, including the heart, liver, spleen, lung, kidney, and brain, were systematically dissected and rinsed with physiological saline to remove external contaminants before being gently dried with filter paper. All collected specimens were rapidly frozen in liquid nitrogen and preserved at −80 °C until further processing. For analytical quantification, tissue homogenates were prepared and processed using protein precipitation methodology prior to HPLC-MS/MS analysis. Tissue concentration calculations were performed according to the following equation:

$$C_{tissue}\left(ng/g\right)=\frac{C_{sample}\left(ng/mL\right)\times V_{homogenate}\left(mL\right)}{M_{tissue}\left(g\right)}$$

To quantify the extent of drug distribution into brain, the brain-to-plasma partition coefficient (Kp) was calculated. Kp is defined as the ratio of the drug concentration in brain to its concurrent concentration in plasma at a given time point. The calculations were performed according to the following equation. This coefficient is particularly used to evaluate the ability of a drug to cross the blood-brain barrier, where a Kp > 1 indicates a higher drug concentration in brain tissue than in plasma

$$Kp=\frac{C_{brain}}{C_{plasma}}$$

#### 2.3.5 Excretion study of ZJCK-6-72 in SD rats

Six male Sprague-Dawley rats received a single oral administration of ZJCK-6-72 (20 mg/kg bodyweight) and were immediately transferred to metabolism cages for individual housing, enabling segregated collection of urinary and fecal outputs. Throughout the 72-h experimental period, all animals were provided with *ad libitum* access to food and water.

Biological specimens were systematically harvested at predetermined intervals: 0–4, 4–8, 8–12, 12–24, 24–36, 36–48, 48–60, and 60–72 h post-dosing. Fecal samples underwent lyophilization followed by mechanical pulverization to achieve homogeneous powders, with exact weights recorded for quantitative analysis. All collected specimens were preserved at −80 °C until subsequent analytical processing.

### 2.4 Metabolic studies

#### 2.4.1 Rat liver microsomes stability study

The metabolic stability of ZJCK-6-72 was evaluated in rat liver microsomes according to established protocols. The incubation system contained 0.1 M phosphate buffer (pH 7.4), NADPH-generating components, and male Sprague-Dawley rat liver microsomes, with the test compound concentration maintained at 1 μM in a final volume of 200 μL. The metabolic reaction was initiated by combining pre-warmed solutions A (NADPH system) and B (microsomes and compound), followed by continuous incubation at 37 °C with agitation. Aliquots were collected at 0, 5, 15, 30, 45, and 60-min intervals, with reactions immediately quenched with four volumes of ice-cold acetonitrile. All experiments included triplicate determinations alongside negative controls without NADPH components and testosterone as a positive control. After centrifugation, supernatants underwent HPLC-MS/MS analysis to quantify residual parent compound.

The residual percentage of ZJCK-6-72 at each interval was determined using peak area ratios relative to the initial time point. Metabolic parameters were derived from the linear regression of the natural logarithm of remaining compound versus incubation time, with the elimination rate constant (k) obtained from the slope. The *in vitro* half-life (t_1_/_2_) and intrinsic clearance (CLᵢ_nt_) were calculated using standard kinetic equations.

$$t_{1/2}=\frac{-0.693}{k}$$

$${CL}_{\mathrm{int}}=\frac{0.693}{t_{1/2}}\times \frac{incubation\;volume\;\left(mL\right)}{microsomes\;weight\;\left(mg\right)}\times Scaling\;Factor$$

#### 2.4.2 CYP450 enzyme metabolic phenotype study

The enzymatic pathways responsible for ZJCK-6-72 metabolism were characterized using human liver microsomes and selective chemical inhibitors. The incubation system contained 0.1 M phosphate buffer (pH 7.4), NADPH-generating components, human liver microsomes (20 mg/mL), and the investigational compound at 1 μM, with specific inhibitors targeting major CYP450 isoforms: α-naphthoflavone (CYP1A2), sertraline (CYP2B6), montelukast (CYP2C8), sulfaphenazole (CYP2C9), nootkatone (CYP2C19), quinidine (CYP2D6), and ketoconazole (CYP3A4). The use of chemical inhibitors in human liver microsomes provides strong evidence for CYP enzyme involvement. Parallel control incubations proceeded without selective inhibitors. All reactions commenced at 37 °C and were terminated with ice-cold acetonitrile following isoform-specific incubation periods (10 min for CYP2C8/2D6/3A4; 20 min for CYP2C9; 30 min for CYP1A2/2B6/2C19). After centrifugation, supernatants underwent HPLC-MS/MS analysis to determine residual parent compound concentrations.

Metabolic rates were calculated from parent compound depletion, with inhibitor groups compared to the control group. The inhibitory effect for each CYP isoform was quantified using standard percentage inhibition calculations.

$$\text{Inhibition}\left(\%\right)=\left(1-\frac{\text{Metabolic}\;\text{Rate}\;\text{in}\;\text{Inhibitor}\;\text{Group}}{\text{Metabolic}\;\text{Rate}\;\text{in}\;\text{Noninhibitor}\;\text{Group}}\right)\times 100\%$$

#### 2.4.3 Inhibition effects of ZJCK-6-72 on CYP450 enzymes

The inhibitory potential of ZJCK-6-72 against seven major human cytochrome P450 enzymes was assessed using pooled human liver microsomal preparations. The incubation system contained 100 μM phosphate buffer (pH 7.4), human liver microsomes (0.2 mg/mL), and the test compound across a concentration range of 0.001–50 μM. A substrate cocktail approach simultaneously evaluated five isoforms: CYP1A2 (phenacetin 40 μM), CYP2C9 (diclofenac 5 μM), CYP2C19 (mephenytoin 50 μM), CYP2D6 (dextromethorphan 5 μM), and CYP3A4 (midazolam 2.5 μM). Separate incubations were conducted for CYP2B6 (bupropion 50 μM), CYP2C8 (amodiaquine 2 μM), and CYP3A4 (testosterone 50 μM) to avoid substrate interference.

Following a 7-min pre-incubation at 38 °C, metabolic reactions were initiated with NADPH (1 mM) and terminated after 10 min using acetonitrile containing internal standards. The assay system was validated with established inhibitors including furafylline (CYP1A2), sulfaphenazole (CYP2C9), N-3-benzylnirvanol (CYP2C19), quinidine (CYP2D6), ketoconazole (CYP3A4), and quercetin (CYP2C8).

#### 2.4.4 *In vivo* metabolites identification and profiling of ZJCK-6-72

Biological specimens including plasma, urine, and fecal matter were obtained from SD rats following single 20 mg/kg bodyweight oral administration of ZJCK-6-72. Plasma samples were proportionally combined based on AUC values to generate time-representative pools, while excreta samples were pooled using equivalent volume ratios across collection periods.

All matrices underwent standardized preparation through protein precipitation with three volumes of chilled acetonitrile. After centrifugation, the resulting supernatants were vacuum-concentrated and reconstituted in 10% acetonitrile-aqueous solution for subsequent analysis. Chromatographic separation was achieved using an ACQUITY UPLC HSS T3 column (2.1 × 100 mm, 1.8 μm) at 25 °C with 0.3 mL/min flow rate and 10 μL injection volume. The 22-min gradient employed 0.1% formic acid in water (A) and acetonitrile (B) as mobile phases.

Mass spectrometric detection utilized a Q-Exactive Plus instrument operating in ESI-positive mode with 80-1200 m/z scan range. Key parameters included: 70,000 full-MS and 17,500 dd-MS (Alzheimer’s Disease Facts and Figures, 2024) resolution; 100/50 m injection times; 1 m/z isolation window; multi-step normalized collision energies (35–65 eV); and 5.0 ppm mass tolerance. System operation was maintained through Xcalibur software with optimized gas flows and lens configurations.

### 2.5 *In vitro* plasma and brain tissue protein binding assay

The unbound fractions of ZJCK-6-72 in rat plasma (f_u,plasma_) and brain tissue (f_u,brain_), were determined using the equilibrium dialysis method. Dialysis membranes were activated prior to use to remove preservatives. Rat brain tissue was homogenized in PBS buffer (pH 7.4) at a ratio of 1:5 (w/v). ZJCK-6-72 was spiked into fresh rat plasma or brain homogenate to achieve a final concentration of 2 μM. An aliquot of the spiked matrix (1 mL) was loaded into the dialysis bag, which was then immersed in a reservoir containing blank PBS (30 mL), maintaining a donor-to-receiver volume ratio of 1:30. The assembly was incubated at 37 °C for 12 h with constant gentle agitation to achieve equilibrium.

Following incubation, aliquots were collected from both the inner compartment and the outer reservoir. To ensure matrix consistency for bioanalysis, blank matrix was added to the buffer samples, and blank PBS buffer was added to the matrix samples. The concentrations of ZJCK-6-72 were quantified using LC-MS/MS method. The unbound fraction in plasma and the apparent unbound fraction in brain homogenate were calculated as the ratio of the drug concentration in the outer buffer to that in the inner matrix compartment. The unbound fraction in brain tissue was calculated by correcting the apparent unbound fraction in brain homogenate for the dilution factor (D = 5). The actual unbound fraction in plasma and brain tissue was using the following equation:

$$f_{u,plasma}=\frac{C_{buffer}}{C_{plasma}}$$

$$f_{u,brain,app}=\frac{C_{buffer}}{C_{brain\;homogenate}}$$

$$f_{u,brain}=\frac{1/D}{\left(\frac{1}{f_{u,brain,app}}-1\right)+\frac{1}{D}}$$

Finally, the unbound brain-to-plasma partition coefficient (K_p,uu_) was calculated as

$$K_{p,uu}=\frac{K_{p}\times f_{u,brain}}{f_{u,plasma}}$$

### 2.6 Acute oral toxicity assessment of ZJCK-6-72

#### 2.6.1 Acute oral toxicity of ZJCK-6-72 (up and down procedure)

The acute toxicity profile of ZJCK-6-72 was characterized following OECD Guideline 425 employing the up-and-down methodology. C57BL/6J mice were subjected to overnight fasting with water available and libitum before compound administration. A sequential dosing regimen was implemented utilizing predefined doses of 55, 82, 120, 175, and 260 mg/kg bodyweight, with the initial dose established through preliminary investigations. Animals received single oral administrations with subsequent dosing decisions guided by preceding survival outcomes. Comprehensive toxicity monitoring was conducted at 30-min, 4-h, 12-h and 24-h intervals post-dosing, followed by daily observations throughout a 14-day period.

#### 2.6.2 Hematoxylin-eosin staining

Histopathological assessment was conducted to identify potential target organ toxicity corresponding to the compound’s substantial volume of distribution and established LD_50_ value. Male C57BL/6J mice were allocated to seven experimental groups (n = 6) comprising vehicle control and six dosage cohorts (500, 350, 245, 170, 120, and 85 mg/kg bodyweight). This extensive dosage range encompassed clearly toxic concentrations exceeding the LD_50_ through subtoxic levels, enabling comprehensive observation of concentration-dependent pathological manifestations. Following single oral administration, animals were monitored for 7 days with survivors euthanized terminally. Subjects succumbing to toxicity or requiring humane endpoints underwent immediate necropsy. Major organs including cardiac, hepatic, splenic, pulmonary and renal tissues were harvested for 10% neutral buffered formalin fixation, followed by standard paraffin embedding, microtome sectioning and hematoxylin-eosin staining. Blinded pathological evaluation was performed by qualified histopathologists.

### 2.7 Data acquisition and analysis

Chromatographic data acquisition and processing utilized Analyst MD (v1.6.2) and MultiQuant MD (v3.0.2) platforms. Quantification of ZJCK-6-72 employed analyte-to-internal standard peak area ratios, with calibration curves generated through linear regression of ratio-to-concentration relationships. Pharmacokinetic parameters were derived via non-compartmental analysis in DAS software (v2.0) using plasma concentration-time profiles. Inhibitory concentration values (IC_50_) were determined through four-parameter logistic curve fitting in XLfit. The median lethal dose (LD_50_) with 95% confidence intervals was calculated using AOT425StatPgm, while additional statistical analyses were conducted in GraphPad Prism (v10.0). All experimental data are expressed as mean ± standard deviation.

## 3 Results and discussion

### 3.1 Pharmacokinetics and bioavailability profile of ZJCK-6-72 in rats

**FIGURE 2:**
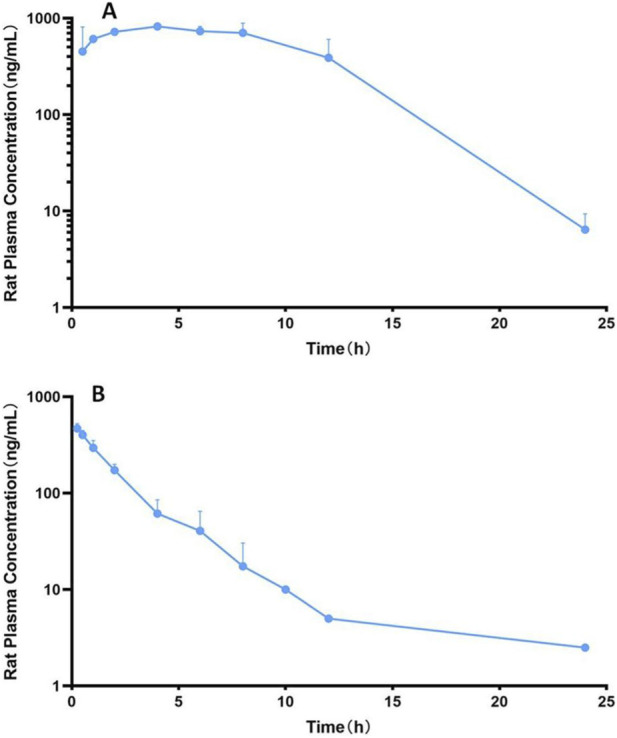
Mean plasma concentration-time curve of ZJCK-6-72 in SD rats after a single **(A)** p.o. (20 mg/kg bodyweight) and **(B)** i.v. (5 mg/kg bodyweight) administration.

The mean plasma concentration-time profiles of ZJCK-6-72 in rats are shown in Figure 2. As shown in Table 1, the PK parameters for each administration route are presented separately. After oral administrations, ZJCK-6-72 was steadily absorbed, with a T_max_ of 3.50 ± 1.76 h. The C_max_ values were 902.32 ± 254.06 ng/mL after oral administration.

**TABLE 1:** Pharmacokinetic parameters of ZJCK-6-72 in rats following intravenous (5 mg/kg bodyweight) and oral (20 mg/kg bodyweight) administration.

Pharmacokinetics parameters	Oral administration	Intravenous administration
Dose (mg/kg bodyweight)	20	5
AUC_0→t_(h*ng/mL)	5819.57 ± 1022.14	1864.52 ± 1022.70
AUC_0→_ ∞ (h*ng/mL)	6076.91 ± 1220.44	1926.16 ± 1007.62
MRT_0-t_(h)	4.46 ± 0.89	2.12 ± 0.72
MRT_0-_ ∞ (h)	4.57 ± 0.80	2.67 ± 1.53
T_1/2_(h)	1.71 ± 1.5	2.14 ± 1.73
T_max_(h)	3.50 ± 1.76	-
C_max_(ng/mL)	902.32 ± 254.06	892.40 ± 434.98
Vd(mL/kg)	-	10.06 ± 9.79
CL(mL/h/kg)	-	3.20 ± 1.47
F(%)	78.03%	

The parameters for each route are presented separately. Absolute oral bioavailability (F) was calculated using dose-normalized AUC, values (F = AUC_*po*_/Dose_*po*_)/(AUC_*iv*_/Dose_*iv*_) ∙100%). Direct comparison of raw values between two dose levels is not intended and would be inappropriate without dose normalization.

ZJCK-6-72 exhibited rapid plasma clearance, with a Cl_int_ of 3.20 ± 1.47 mL/kg/h after i.v. administration and a short elimination half-life, with a t_1/2_ value of 1.714 ± 1.524 h for oral, and 2.141 ± 1.732 h for i.v. administration, suggesting a low potential for accumulation in the body. The apparent volume of distribution (Vd) was high, with a value of 10.06 ± 9.79 mL/kg after i.v. administration, indicating extensive tissue penetration of ZJCK-6-72 within the body. The mean absolute oral bioavailability was 78.03%, demonstrating excellent oral absorption. This value is substantially higher than the average for orally administered small molecules and represents a significant optimization over the bioavailability reported for ZJCK-6-46. The results support the potential for further development as an oral formulation.

It should be noted that the calculation of absolute oral bioavailability (78.03%) via dose-normalized AUC comparison assumes linear pharmacokinetics. Although a formal dose-proportionality study has not yet been performed, the overlapping concentration ranges achieved with these two doses support the validity of this comparison.

### 3.2 Tissue distribution of ZJCK-6-72 in rats

**FIGURE 3:**
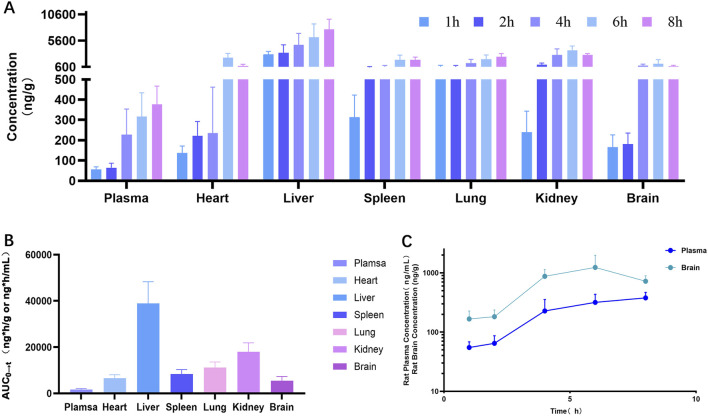
Tissue distribution characteristics of ZJCK-6-72 in rats following a single oral administration (20 mg/kg bodyweight). **(A)** Tissue concentration of ZJCK-6-72 at 1, 2, 4, 6, and 8 h post-dosing; **(B)** Tissue exposure (AUC_0→t_) of ZJCK-6-72; **(C)** Comparative concentration-time profiles of ZJCK-6-72 in plasma and brain tissue, demonstrating sustained brain exposure. (Data are expressed as Mean ± SD, n = 5 per time per group).

Tissue and plasma concentrations of ZJCK-6-72 were determined at multiple time points after a single oral dose of 20 mg/kg bodyweight, which the same dose used for the oral pharmacokinetic and excretion studies to maintain experimental consistency. As illustrated in Figure 3A, the compound was rapidly distributed into most tissues. According to the total concentration AUC_0-t_ in Figure 3B, from high to low, tissues were the liver > kidney > lungs > spleen > heart > brain. Furthermore, the comparative concentration-time profiles of ZJCK-6-72 in plasma and brain tissue are shown in Figure 3C, demonstrating sustained brain exposure over 8 hours post-dose.

The most significant advancement of ZJCK-6-72 lies in its exceptional brain penetration capability, a critical prerequisite for targeting DYRK1A within the central nervous system. As shown in Table 2, after a single oral administration (20 mg/kg bodyweight) in SD rats, ZJCK-6-72 achieved a remarkably high K_p_ value, ranging from 1.92 to 4.63 across different time points. This represents a more than 20-fold increase in brain exposure compared to ZJCK-6-46, clearly indicating that the strategic structural modifications in ZJCK-6-72 successfully addressed the key limitation of its predecessor. The compound’s distribution profile, characterized by high brain penetration, suggests potential for pharmacological activity in the central nervous system, which may translate into therapeutic effects in Alzheimer’s disease models. These findings establish the central nervous system as the primary therapeutic target for ZJCK-6-72, confirming its effective blood-brain barrier crossing capability.

**TABLE 2:** Brain/Plasma concentration ratio of ZJCK-6-72 after 20 mg/kg bodyweight oral administration, and K_p_ (brain/plasma) value of ZJCK-6-46 after 10 mg/kg bodyweight oral administration.

Time point (h)	Concentration of ZJCK-6-72		K_p_ (brain/plasma)	
	Plasma (ng/mL)	Brain (ng/g)	ZJCK-6-72	ZJCK-6-46(Chen et al., 2024)
1	54.91 ± 13.56	166.28 ± 60.51	2.98 ± 0.62	0.08 ± 0.01
2	64.46 ± 22.31	181.73 ± 54.15	3.04 ± 0.85	0.14 ± 0.06
4	227.46 ± 126.54	873.20 ± 259.10	4.63 ± 2.04	0.19 ± 0.12
6	317.04 ± 117.31	1234.25 ± 736.37	3.76 ± 2.08	0.14 ± 0.04
8	377.44 ± 90.05	721.68 ± 168.79	1.92 ± 0.11	-

Tissue distribution analysis revealed predominant hepatic accumulation corresponding to its major metabolic pathway, which is consistent with the liver’s physiological role in drug metabolism and excresion. This hepatic accumulation is supported by the CYP phenotyping data and the excretion data presented in Section 3.4 and 3.5.2. While high hepatic exposure might theoretically raise concerns about first-pass extraction compromising systemic availability, the high oral bioavailability directly demonstrates that presystemic metabolism is not a limiting factor. Furthermore, the parallel brain liver and brain concentrations from 4-8 h post-dose (Figure 3A) reflects distribution equilibrium with the systemic circulation rather than hepatic sequestration. Most importantly, the unbound brain-to-plasma partition coefficient significantly exceeds unity, as discussed in the following section, providing definitive evidence that brain penetration is not compromised but actively enhanced. Rapid widespread distribution suggests extensive tissue perfusion and potential transporter involvement. The collective data substantiate that ZJCK-6-72 achieves adequate brain exposure to mediate its intended anti-Alzheimer effects.

It is noted that plasma concentration in the tissue distribution study showed a delayed peak compared to the dedicated PK study. This discrepancy likely arises from inter-individual variability and the sparse sampling design in the tissue distribution study, whereas the intensive sampling in the PK study provides a more accurate estimation of absorption kinetics.

### 3.3 Plasma protein binding and unbound brain partition coefficient

The protein binding of ZJCK-6-72 was assessed in rat plasma and brain homogenate. As shown in Table 3, ZJCK-6-72 exhibited high binding to both matrices, with unbound fractions (f_u_) of 3.30% ± 1.03% in plasma and 3.41% ± 0.30% in brain tissue. Despite the extensive protein binding, the unbound brain-to-plasma partition coefficient (Kp,uu), calculated based on the AUC_0→t_ ratio (Kp = 3.39), was determined to be 3.50, which significantly greater than unity, indicates that the unbound drug concentration in the brain effectively exceeds that in the plasma, suggesting efficient BBB penetration and potential involvement of active uptake mechanisms.

**TABLE 3:** Protein binding and brain penetration parameters of ZJCK-6-72 in rats (n = 3).

Parameter	Value
Total brain/Plasma ratio (K_p, AUC_)	3.39
Fraction unbound in plasma (f_u,plasma_)	3.30% ± 1.03%
Fraction unbound in brain (f_u, brain_)	3.41% ± 0.30%
Unbound brain/Plasma partition coefficient(K_p,uu_)	3.50

According to established classification criteria, a Kp,uu value greater than 2 indicates tissue enrichment (Orozco et al., 2020; Rankovic, 2015). Therefore, the observed Kp,uu of 3.50 strongly suggests the involvement of active uptake transporters at the blood-brain barrier, facilitating the entry of ZJCK-6-72 against the concentration gradient.

### 3.4 Urine and feces excretion of ZJCK-6-72

**FIGURE 4:**
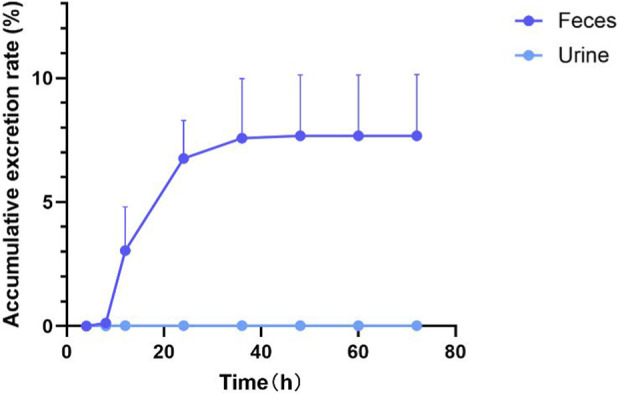
Cumulative excretion rate of ZJCK-6-72 in SD rats.

As shown in Figure 4, ZJCK-6-72 underwent rapid elimination predominantly through the fecal route, with 7.69% ± 2.47% of the administered dose recovered as parent compound within 72 h. Excretion occurred primarily during the initial 48-h period, where fecal elimination accounted for 7.67% ± 0.02% of the dose versus minimal urinary recovery of 0.02% ± 0.01%. These findings indicate that fecal elimination, likely mediated primarily by biliary excretion of the parent drug is the major clearance route. The low cumulative recovery of unchanged compound indicates extensive *in vivo* metabolism, supporting metabolite profiling data that suggest rapid biotransformation and subsequent elimination of metabolic products through unidentified pathways.

The low recovery of unchanged parent drug in feces despite high oral bioavailability suggests that the majority of the absorbed dose is metabolized prior to excretion. The predominance of fecal excretion, coupled with the observed metabolic profile, strongly points to biliary excretion of phaseI/II metabolites as the primary elimination route. Furthermore, as illustrated in the following Section 3.5.4, the presence of glucuronide conjugates (M7, M11) in feces raises the possibility of enterohepatic recirculation, which could contribute to the prolonged exposure observed in some tissues. Direct intestinal secretion of the parent drug cannot be ruled out, but is considered a minor pathway based on the tissue distribution.

### 3.5 Metabolism study of ZJCK-6-72

#### 3.5.1 Metabolic stability in liver microsomes

Metabolic stability assessment of ZJCK-6-72 across species revealed intrinsic clearance values of 80.27 and 76.42 μL/min/mg protein in human and rat liver microsomes, respectively, with corresponding half-lives of 21.66 and 32.50 min. The metabolic profile, while distinct from the predecessor, correlated with the compound’s high lipophilicity. Such structural optimization typically introduces conformational flexibility and reduced lipophilicity that consequently create vulnerable sites for cytochrome P450-mediated oxidation (Rankovic, 2015). This strategic compromise successfully achieved the primary objective of significantly improved brain exposure while maintaining adequate systemic coverage through high oral bioavailability (78.03%). The resulting pharmacokinetic profile emphasizing effective central nervous system delivery over extended plasma circulation aligns with established requirements for neurotherapeutic agents and justifies continued development of ZJCK-6-72.

#### 3.5.2 CYP450 phenotype of ZJCK-6-72

The CYP phenotyping study using selective inhibitors revealed a multi-enzyme clearance mechanism for ZJCK-6-72. Seven major CYP450 enzymes, including CYP3A4, CYP2C9, CYP2D6, CYP2C8, CYP1A2, CYP2C19 and CYP2B6, were profiled to determine which enzymes were responsible for the metabolism of ZJCK-6-72 using human liver microsomes (HLMs). The remaining ZJCK-6-72 in HLMs following a 5, 10, 20, and 30 min incubation without inhibitors was 75.41%, 57.64%, 40.21%, and 38.79% respectively. In the presence of selective inhibitors, the remaining amount of ZJCK-6-72 was greater than that without inhibitors, as presented in Table 4. The inhibition rates of CYP1A2, CYP2C19, and CYP2B6 were 86.6%, 82.39%, and 71.40% respectively, while other CYPs were less than 60%. This reliance on multiple CYP isoforms may be clinically advantageous, as it reduces the risk of dramatic exposure changes due to genetic polymorphisms in a single enzyme or co-administration of specific enzyme inhibitors. However, given the significant involvement of CYP2B6, future clinical studies should also consider the potential impact of CYP2B6 polymorphisms and variability in expression levels.

**TABLE 4:** Integrated summary of *in vitro* CYP metabolic phenotype and inhibition profile of ZJCK-6-72.

CYPs	Metabolic phenotype			Inhibition	
	Selective inhibitor	Incubation time (min)	Inhibition rate (%)	Probe substrate	IC50 (μM)
CYP3A4	Ketoconazole	10	42.30	Midazolam	15.69
				Testosterone	1.74
CYP2C9	Sulfaphenazole	20	23.36	Diclofenac	0.45
CYP2D6	Quinidine	10	48.34	Dextromethorphan	5.17
CYP2C8	Montelukast	5	0.14	Amodiaquine	3.42
CYP1A2	α-Naphthoflavone	30	86.60	Phenacetin	1.84
CYP2C19	Nootkatone	30	82.39	Mephenytoin	1.76
CYP2B6	Sertraline	30	71.40	Bupropion	>50

The inhibition data corroborate the phenotype results, showing potent inhibition of CYP1A2 and CYP2C19, the very enzymes responsible for its metabolism. This presents a classic auto-inhibition scenario that may lead to non-linear pharmacokinetics at higher doses.

#### 3.5.3 Inhibition effects of ZJCK-6-72 on CYP450 enzymes

The potential for drug-drug interactions mediated by cytochrome P450 (CYP) inhibition is a critical consideration in drug development. Inhibition of these enzymes can lead to clinically significant drug-drug interactions, altering the pharmacokinetics of co-administered drugs.

**FIGURE 5:**
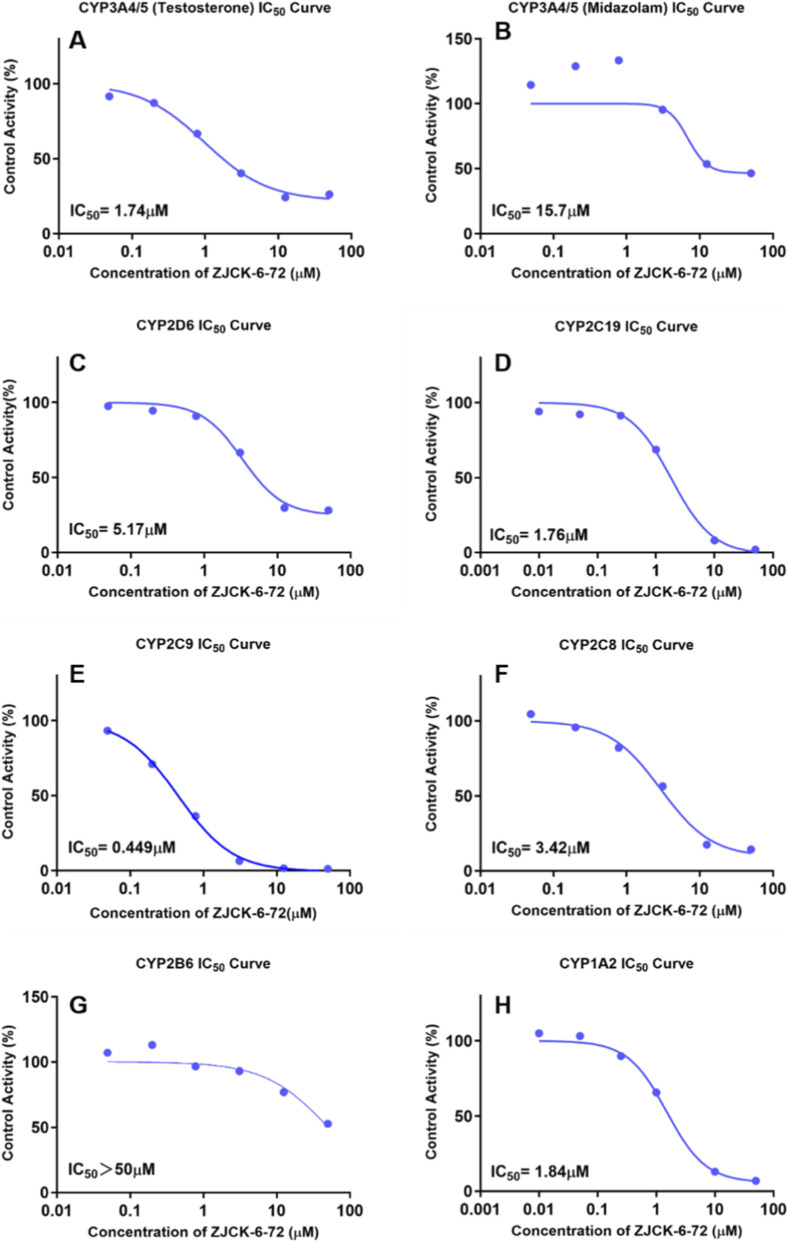
The inhibitory effects of **(A)** CYP3A4-Testosterone; **(B)** CYP3A4-Midazolam; **(C)** CYP2D6; **(D)** CYP2C19; **(E)** CYP2C9; **(F)** CYP2C8; **(G)** CYP2B6; **(H)** CYP1A2 activity by ZJCK-6-72.

The potential for ZJCK-6-72 to cause DDI via mechanism based CYP450 inhibition was evaluated *in vitro*. As summarized in Table 4 and Figure 5, ZJCK-6-72 indicates a broad potential for DDI, with risk levels varying significantly across isoforms. The most potent inhibition was observed against CYP2C9, with an IC_50_ of 0.45 μM. Considering the maximal plasma concentration (Cmax) of approximately 2.14 μM at the effective dose, the [I]/IC_50_ ratio reaches 4.7, flagging a critical risk for interactions with narrow therapeutic index substrates like warfarin or phenytoin (Ashrafpour and Ashrafpour, 2025; Steulet et al., 2024; Fitzgerald and Howes, 2016). Similarly, ZJCK-6-72 showed potent inhibition of CYP3A4 when probed with testosterone (IC_50_ = 1.74 μM), suggesting it could alter the metabolism of steroid-like substrates. Interestingly, the inhibition was much weaker against midazolam (IC_50_ = 15.7 μM), indicating substrate-dependent inhibition (Lee et al., 2012; VandenBrink et al., 2011; Do et al., 2011). This discrepancy often arises from the existence of multiple binding regions within the large CYP3A4 active site, suggesting ZJCK-6-72 may preferentially bind to the steroid-binding pocket. ZJCK-6-72 also inhibited its own metabolizing enzymes, CYP1A2 (IC_50_ = 1.84 μM) and CYP2C19 (IC_50_ = 1.76 μM), with [I]/IC_50_ ratio exceeding unity. This overlap of substrate and inhibitor specificity points to a mechanism of reversible metabolic auto-inhibiton (Mohamed et al., 2017; Wu et al., 2020; Duan et al., 2019), which may partially explain the high oral bioavailability (78.03%) despite the compound’s high lipophilicity, as presystemic metabolism could be saturated or inhibited during absorption. While this did not prevent rapid clearance in the single-dose study, it warrants close monitoring in multiple-dose escalation studies, as it could lead to non-linear pharmacokinetics and accumulation over time. Moderate inhibitory effects were observed for CYP2C8 (IC_50_ = 3.42 μM) and CYP2D6 (IC_50_ = 5.17 μM). Although less concerning than CYP2C9, the potential for interactions cannot be ruled out if systemic accumulation occurs.

Collectively, the inhibition data provide mechanistic validation for previous metabolic identification of CYP1A2, 2C19 and 2B6 as primary metabolizing enzymes. The simultaneous substrate and inhibitor relationship with these cytochrome P450 isoforms necessitates clinical drug-drug interaction studies, particularly for concomitant medications metabolized by CYP2C9, 2C19, 1A2 and 3A4, to establish safe administration parameters.

#### 3.5.4 Metabolite identification and profiling in plasma, urine, and feces

**FIGURE 6:**
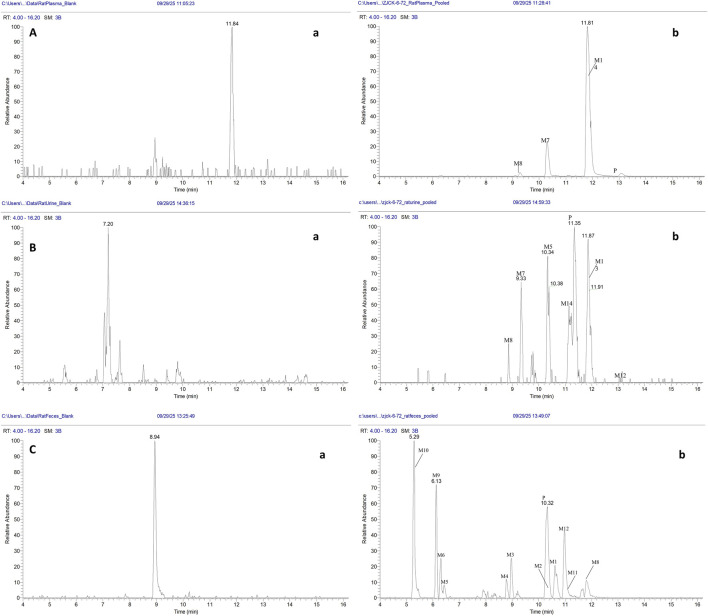
Representative extracted ion chromatograms (EICs) of ZJCK-6-72 and its metabolites in rat biological matrices, with blank matrix controls. **(a)** Selected ion chromatogram of ZJCK-6-72 in pooled rat matrices **(A)**. Plasma, **(B)**. Urine, **(C)**. Feces after oral administration; **(b)** Selected ion chromatogram of blank matrices **(A)**. Plasma, **(B)**. Urine, **(C)**. Feces.

Representative extracted ion chromatograms (EICs) of ZJCK-6-72 and its identified metabolites in rat plasma, urine, and feces are shown in Figure 6. The chromatograms confirm the presence of the parent compound and all 14 metabolites (M1-M14) across different biological matrices, with retention times consistent with those listed in Table 5.

**TABLE 5:** Observed metabolites of ZJCK-6-72 in rat plasma, urine and feces after oral administration.

Metabolite	Metabolic pathway	RT (min)	Relative peak area abundance (%)		
			Plasma	Urine	Feces
M1	*N*-dealkylation	5.29	ND	ND	23.0
M2	Mono-oxygenation and *N, O*-dealkylation	6.14	D	ND	13.7
M3	*N, O*-dealkylation	6.30	0.2	ND	5.2
M4	Mono-oxygenation and *N, O*-dealkylation	6.43	ND	ND	1.8
M5	Mono-oxygenation	8.78	D	2.4	2.6
M6	Di-oxygenation	8.97	D	ND	5.3
M7	Mono-oxygenation and glucuronidation	9.31	1.1	10.4	0.1
M8	Mono-oxygenation	10.31	14.7	17.8	18.4
M9	Mono-oxygenation and sulfation	10.35	ND	ND	3.6
M10	Di-oxygenation	10.63	0.1	D	7.8
M11	Glucuronidation	10.97	0.1	ND	11.9
M12	Oxidation	11.10	0.5	23.0	2.1
M13	Di-oxygenation	11.36	0.1	21.0	ND
P (ZJCK-6-72)	-	11.83	81.5	25.4	4.5
M14	Mono-oxygenation	13.28	1.7	D	ND

P: parent; D: detected; ND: not detected; metabolites with a relative peak area abundance of less than 1% were not marked in the following selected ion chromatograms; the relative abundance of the parent and metabolites was calculated based on their mass chromatographic peak areas.

As shown in Table 5, 14 metabolites (M1-M14) of ZJCK-6-72 were identified in rat plasma, urine, and feces, including mono-oxygenation metabolites (M2, M4, M5, M7, M8, M9, M14), di-oxygenation metabolites (M6, M10, M13), oxidation metabolites (M12) N/O-dealkylation metabolites (M1, M2, M3, M4), glucuronidation metabolites (M7, M11), and sulfation metabolites (M9). The relative abundance of the parent drug and its metabolites varied significantly across the biological matrices.

**FIGURE 7:**
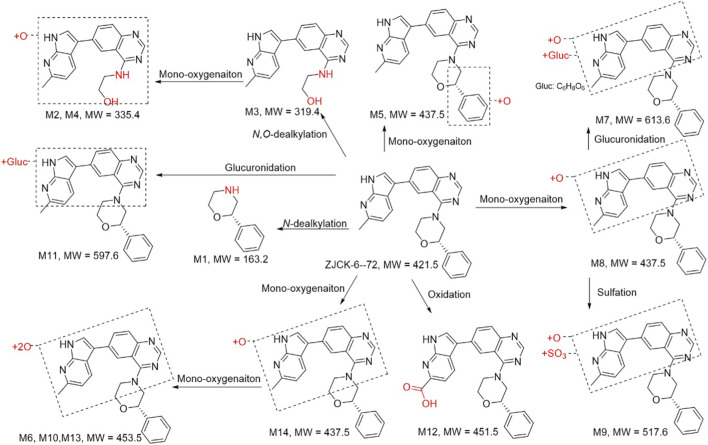
Proposed metabolic pathways of ZJCK-6-72 in rat plasma, urine and feces after oral administration.

As shown in Figure 7 and Table 5, the parent drug (ZJCK-6-72) was the predominant circulating component, accounting for 81.5% of the total detected peak area. The most abundant metabolite in plasma was M8 (mono-oxygenation, 14.7%). Trace amounts of other metabolites, such as M7 (glucuronide conjugate), were also detected. The metabolic profile in urine was more diverse. While the parent compound remained substantial (25.4%), metabolites M12 (oxidation, 23.0%) and M13 (di-oxygenation, 21.0%) were highly abundant. The glucuronide conjugate M7 represented a major urinary metabolite at 10.4% of administered dose. Fecal samples contained minimal parent compound (4.5%) alongside complex metabolic products, predominantly featuring M1 (N-dealkylation, 23.0%), M2 (mono-oxygenation with N,O-dealkylation, 13.7%), and M11 (glucuronidation, 11.9%).

These findings demonstrate comprehensive biotransformation of ZJCK-6-72 in rats. The primary metabolic routes were: phase I reactions, including Mono-oxygenation (M5, M8, M14) and di-oxygenation (M6, M10, M13), were prevalent, alongside various dealkylation reactions (M1, M2, M3). As for phase II conjugation, glucuronidation (M7, M11) and sulfation (M9) were identified, facilitating the elimination of oxidized metabolites, particularly in urine and feces. In conclusion, ZJCK-6-72 is extensively metabolized *in vivo*, with oxidation and subsequent conjugation being the dominant clearance mechanisms. The parent compound is the major drug-related material in systemic circulation, while metabolites are the primary forms excreted in urine and feces.

### 3.6 Acute toxicity study of ZJCK-6-72

Acute oral toxicity assessment of ZJCK-6-72 in male C57BL/6J mice was performed following OECD Test Guideline 425 (UDP, 2008). The study yielded an LD_50_ value of 233.9 mg/kg bodyweight with 95% confidence limits spanning 175–260 mg/kg bodyweight. According to the Globally Harmonized System classification criteria, this toxicity profile categorizes the compound under GHS Category III, consistent with moderate acute toxicity potential (GHS, 2024). The narrow confidence interval indicates high reliability for the calculated LD_50_ value. Animals administered doses exceeding 175 mg/kg bodyweight demonstrated acute toxic manifestations including reduced mobility, sedation, respiratory distress, righting reflex loss and motor abnormalities, manifesting predominantly within 12 h after administration.

**FIGURE 8:**
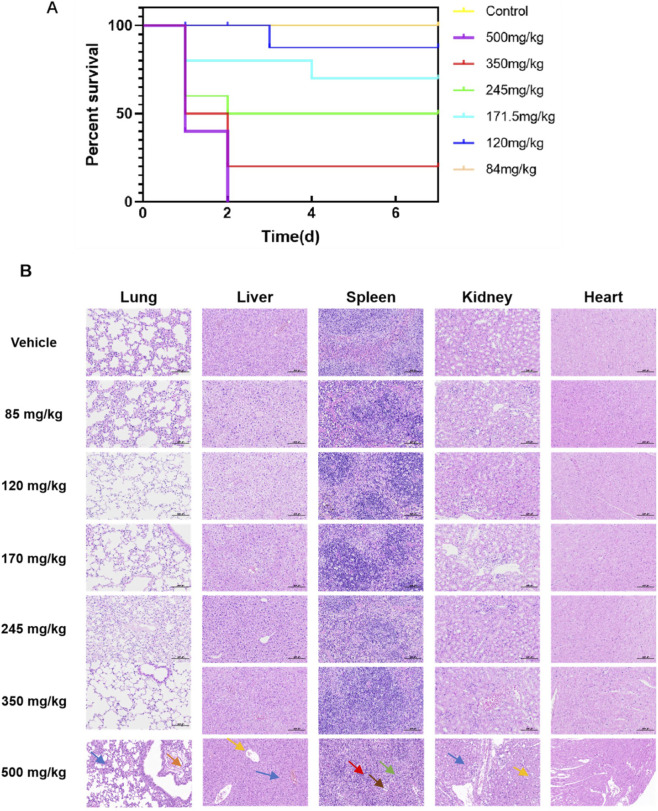
Acute oral toxicity assessment of ZJCK-6-72 in C57BL/6J mice. **(A)** Survival curves of mice following a single oral administration of ZJCK-6-72 at various doses. **(B)** Representative histopathological images of major organs from mice administered a single oral dose of ZJCK-6-72. Tissues were stained with hematoxylin and eosin (H&E); scale bar = 100 μm. Mice administered with 500 mg/kg bodyweight ZJCK-6-72 showed severe hepatocellular damage, including marked hepatocellular edema (blue arrows), microvascular steatosis, and focal necrotic areas (yellow arrows) was observes. The kidney exhibited substantial tubular injury characterized by epithelial edema (blue arrows) and cytoplasmic vacuolization (yellow arrows). Splenic alterations included sinus dilation (brown arrows), white pulp lymphocyte necrosis (red arrows), and red pulp inflammatory infiltration (green arrows). The lung showed mild septal thickening and perivascular edema (orange arrows). The lung showed mild septal thickening and perivascular edema (orange arrows), while the heart displayed minimal pathological involvement, establishing it as a non-target organ under acute exposure conditions.

According to Figure 8, hepatic tissue exhibited the most severe damage after oral administration of 500 mg/kg bodyweight of ZJCK-6-72, with evident hepatocellular edema, microvascular steatosis, and focal necrotic regions. Renal pathology showed significant tubular injury with epithelial edema and cytoplasmic vacuolization. Splenic alterations included white pulp lymphocyte necrosis and red pulp inflammatory infiltration, indicating potential immunotoxicity. Pulmonary changes remained moderate, with mild septal thickening and perivascular edema, while cardiac tissue displayed minimal pathological involvement, confirming its non-target status under acute exposure conditions.

## 4 Conclusion

This comprehensive ADMET study demonstrates that ZJCK-6–72 has overcome the critical liability of poor brain penetration associated with its predecessor, ZJCK-6-46. The compound exhibits a favorable pharmacokinetic profile characterized by high oral bioavailability (78.03%), rapid absorption, and extensive tissue distribution.

The most significant finding is the dramatic enhancement in CNS delivery. ZJCK-6-72 achieved a greater than 20-fold increase in total brain exposure (Kp) compared to ZJCK-6-46. More importantly, the high unbound brain-to-plasma partition coefficient (Kp,uu = 3.50) provides definitive evidence that the structural optimization successfully improved intrinsic BBB permeability. The fact that K_p,uu_ significantly exceeds unity strongly suggests the involvement of active uptake transport mechanisms, ensuring therapeutically relevant unbound drug concentrations in the brain.

Metabolic characterization revealed that ZJCK-6-72 is primarily cleared via CYP1A2 and CYP2C19. The identification of ZJCK-6-72 as both a substrate and an inhibitor of these enzymes highlights a mechanism of metabolic auto-inhibition, which may influence dose-linearity and warrants consideration in future clinical dosing strategies. Despite these complex metabolic interactions and broad tissue distribution, the compound demonstrated an acceptable acute safety margin.

Collectively, these data validate ZJCK-6-72 as a potent, orally bioavailable, and brain-penetrant DYRK1A inhibitor. Its superior unbound brain exposure positions it as a highly promising lead candidate for the treatment of AD, justifying its advancement into further preclinical development.

## Data Availability

The original contributions presented in the study are included in the article/Supplementary Material, further inquiries can be directed to the corresponding authors.
