# Supplementary material for: Pharmacokinetics, distribution, metabolism, excretion and safety characterization of ZJCK-6-72: novel DYRK1A inhibitor with optimized brain exposure for Alzheimer’s disease therapy
Source: Front Pharmacol. 2026 May 1;17:1792258. doi: 10.3389/fphar.2026.1792258 (PMC13175962; doi:10.3389/fphar.2026.1792258)
Supplement: Supplementary file 1 [file DataSheet1.docx]

**Supplementary Material**

**Section 1:** Summary of method validation of the quantitation of ZJCK-6-72 in rat samples.

**Concentration of QC samples:** 12.5 ng/mL (LLOQ), 25 ng/mL (LQC), 800 ng/mL (MQC), 1600 ng/mL (HQC), 8000 ng/mL (5× dilution QC), 16000 ng/mL (10× dilution QC).

**Linearity range:** 12.5 ng/mL~2000 ng/mL

1. **Selectivity**

No significant interference for either ZJCK-6-72 or IS was observed in any of the six lots of blank samples used for the selectivity test.


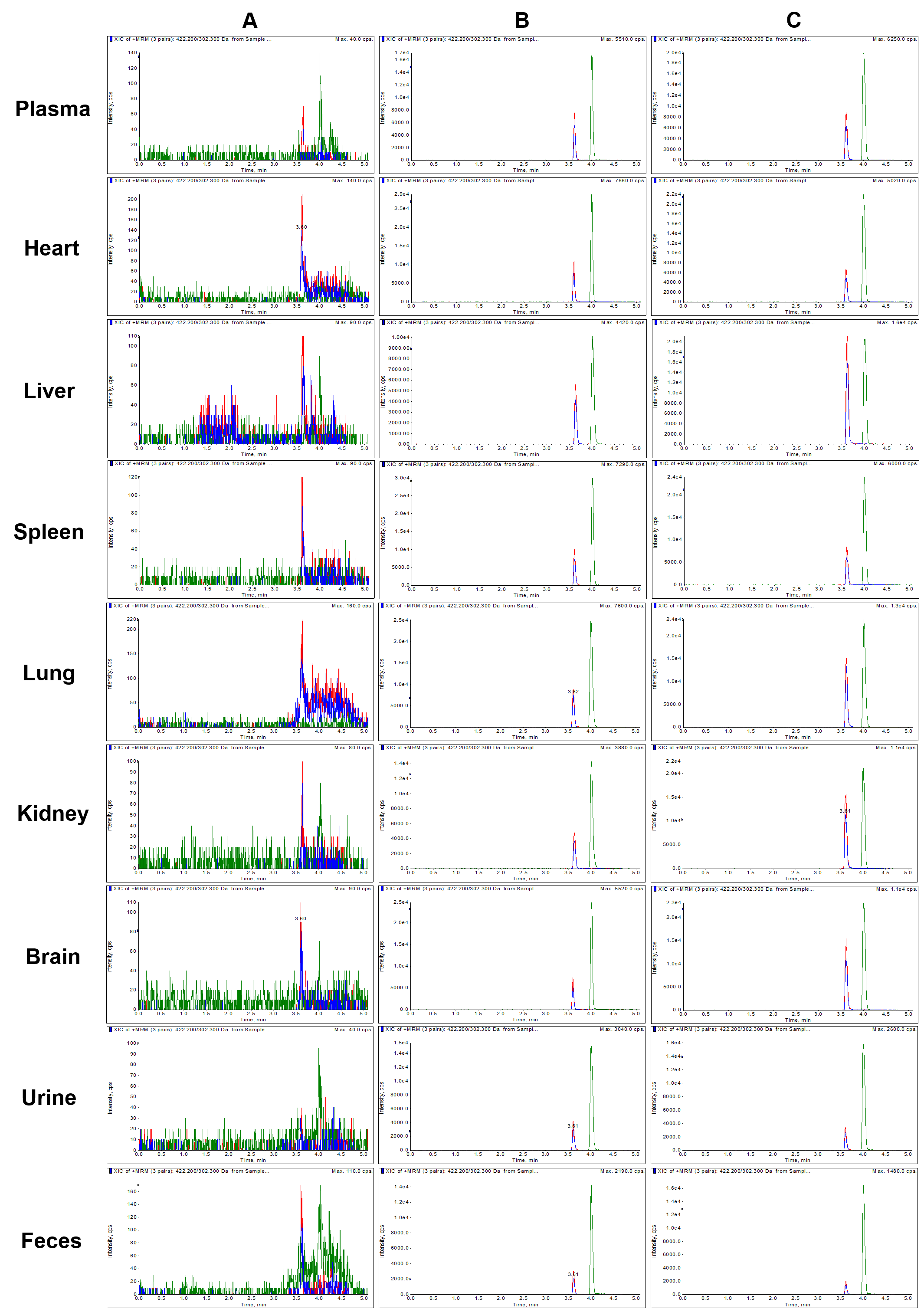


**Figure S1** Representative MRM chromatograms of ZJCK-6-72 and IS in rat matrix samples. A. blank matrix samples; B. blank matrix spiked with ZJCK-6-72 (12.5 ng/mL) and IS (200 ng/mL); C. sample from a rat 1 h after oral administration of ZJCK-6-72 at the dose of 20 mg/kg.

1. **Calibration curves of ZJCK-6-72 quantitation in each sample:**

| **Sample** | **Calibration curve** | **r^2^** |
| --- | --- | --- |
| Plasma | y=121.07x＋1212.54 | 0.9918 |
| Brain | y=136.75x＋17991.89 | 0.9904 |
| Heart | y=212.16x＋19132.37 | 0.9939 |
| Liver | y=118.22x＋18165.82 | 0.9986 |
| Spleen | y=189.39x＋18582.68 | 0.9955 |
| Lung | y=135.31x＋11825.91 | 0.9945 |
| Kidney | y=116.65x＋12987.45 | 0.9900 |
| Urine | y=115.25x＋11207.26 | 0.9922 |
| Feces | y=144.71x＋14120.87 | 0.9900 |

1. **Carryover:**

No significant carryover was observed in the matrix blank injected right after the ULOQ standard.

1. **Accuracy and precision：**

| **Sample** | **Conc.** | **Tested Conc.** | **RE** | **Inter-batch RSD** | **Intra-batch RSD** |
| --- | --- | --- | --- | --- | --- |
| Plasma | LLOQ | 12.36±0.10 | −0.05±2.17 | 0.81 | 1.78 |
|  | LQC | 24.85±1.03 | −0.85±1.23 | 4.14 | 2.67 |
|  | MQC | 806.17±18.09 | 11.00±1.04 | 2.24 | 5.68 |
|  | HQC | 1776.83±17.70 | −0.78±2.26 | 1.00 | 5.86 |
| Brain | LLOQ | 12.08±0.54 | −3.33±4.32 | 4.47 | 11.09 |
|  | LQC | 23.07±0.48 | −7.73±1.91 | 2.07 | 12.74 |
|  | MQC | 825.50±30.96 | 3.19±3.87 | 3.75 | 12.89 |
|  | HQC | 1538.33±71.51 | −3.85±4.47 | 4.65 | 13.87 |
| Heart | LLOQ | 12.05±0.52 | −3.58±4.20 | 4.36 | 11.09 |
|  | LQC | 25.11±1.04 | −0.44±4.17 | 4.15 | 12.74 |
|  | MQC | 791.18±7.19 | −1.10±0.90 | 0.91 | 12.89 |
|  | HQC | 1450.17±25.78 | −9.36±1.61 | 1.78 | 13.87 |
| Liver | LLOQ | 12.06±0.30 | −3.56±2.37 | 2.40 | 2.86 |
|  | LQC | 25.02±0.48 | 0.09±1.92 | 1.89 | 2.89 |
|  | MQC | 835.92±15.77 | 4.49±1.97 | 1.85 | 3.04 |
|  | HQC | 1611.83±11.88 | 0.74±0.74 | 0.73 | 1.25 |
| Spleen | LLOQ | 12.34±0.66 | −1.32±5.28 | 5.35 | 4.88 |
|  | LQC | 25.91±1.33 | 3.63±5.31 | 5.12 | 5.41 |
|  | MQC | 856.18±15.77 | 7.02±1.97 | 1.84 | 2.58 |
|  | HQC | 1603.33±33.40 | 0.21±2.09 | 2.08 | 2.32 |
| Lung | LLOQ | 13.45±0.32 | 7.63±2.58 | 2.40 | 5.01 |
|  | LQC | 26.09±0.66 | 4.36±2.65 | 2.54 | 3.61 |
|  | MQC | 859.68±38.41 | 7.46±4..80 | 4.47 | 4.71 |
|  | HQC | 1736.80±20.36 | 8.55±1.27 | 1.17 | 2.40 |
| Kidney | LLOQ | 13.04±0.59 | 5.80±5.60 | 5.29 | 4.52 |
|  | LQC | 26.00±1.45 | −0.22±6.84 | 6.85 | 5.56 |
|  | MQC | 848.89±19.52 | 7.53±1.60 | 1.49 | 2.30 |
|  | HQC | 1684.67±39.95 | 3.35±3.01 | 2.91 | 2.37 |
| Urine | LLOQ | 12.43±0.75 | 4.04±5.32 | 5.12 | 6.06 |
|  | LQC | 25.87±0.77 | 5.16±3.46 | 3.29 | 2.98 |
|  | MQC | 811.38±38.86 | 0.71±2.90 | 2.88 | 12.89 |
|  | HQC | 1728.05±41.76 | 10.38±0.87 | 0.79 | 13.87 |
| Feces | LLOQ | 12.30±0.72 | 3.73±4.24 | 4.09 | 5.88 |
|  | LQC | 25.95±0.86 | 3.97±2.57 | 2.48 | 3.31 |
|  | MQC | 824.17±50.02 | 3.17±5.36 | 5.20 | 6.07 |
|  | HQC | 1648.61±35.34 | 4.70±1.50 | 1.43 | 2.14 |

1. **Recovery**

| **Sample** |  | **ZJCK-6-72** | | | **IS** |
| --- | --- | --- | --- | --- | --- |
|  |  | **LQC** | **MQC** | **HQC** |  |
| Plasma | Mean | 91.32±4.14 | 107.77±1.30 | 103.38±3.97 | 101.10±5.04 |
|  | RSD | 4.54 | 1.21 | 3.84 | 4.97 |
| Brain | Mean | 116.43±6.77 | 111.90±3.86 | 109.49±3.54 | 87.59±3.11 |
|  | RSD | 5.82 | 3.45 | 3.23 | 3.55 |
| Heart | Mean | 99.35±6.22 | 106.92±2.05 | 103.04±2.28 | 87.59±3.11 |
|  | RSD | 6.27 | 1.92 | 2.21 | 3.55 |
| Liver | Mean | 82.34±4.15 | 93.57±2.08 | 92.03±1.43 | 79.48±2.19 |
|  | RSD | 5.04 | 2.23 | 4.55 | 2.75 |
| Spleen | Mean | 115.66±11.08 | 108.72±1.57 | 108.76±4.46 | 106.94±3.08 |
|  | RSD | 9.58 | 1.44 | 4.10 | 2.88 |
| Lung | Mean | 97.78±6.70 | 100.71±3.70 | 104.07±2.38 | 93.03±4.36 |
|  | RSD | 6.85 | 3.67 | 2.28 | 4.69 |
| Kidney | Mean | 89.73±3.89 | 92.45±3.11 | 94.12±4.35 | 107.46±4.51 |
|  | RSD | 4.33 | 3.37 | 4.62 | 4.20 |
| Urine | Mean | 94.25±5.36 | 96.44±1.48 | 104.36±3.48 | 99.46±4.23 |
|  | RSD | 5.69 | 1.54 | 3.34 | 4.25 |
| Feces | Mean | 103.40±5.15 | 99.78±4.07 | 101.83±2.83 | 97.17±1.40 |
|  | RSD | 4.98 | 4.08 | 2.78 | 1.44 |

1. **Matrix effects (IS normalized)**

| **Sample** |  | **ZJCK-6-72** | | **IS** |
| --- | --- | --- | --- | --- |
|  |  | **LQC** | **HQC** |  |
| Plasma | Mean | 94.45±7.41 | 92.46±5.89 | 87.35±2.59 |
|  | CV, % | 7.84 | 6.37 | 2.71 |
| Brain | Mean | 97.45±3.26 | 92.46±5.89 | 87.35±2.59 |
|  | CV, % | 3.35 | 6.38 | 2.71 |
| Heart | Mean | 99.33±6.13 | 102.09±6.89 | 87.35±2.59 |
|  | CV, % | 6.17 | 6.75 | 2.71 |
| Liver | Mean | 91.17±9.75 | 106.94±2.40 | 102.01±3.69 |
|  | CV, % | 10.69 | 2.25 | 3.62 |
| Spleen | Mean | 90.42±6.68 | 102.22±3.70 | 102.22±3.70 |
|  | CV, % | 7.38 | 3.62 | 3.62 |
| Lung | Mean | 86.59±3.45 | 105.44±5.02 | 103.61±3.23 |
|  | CV, % | 3.98 | 4.76 | 3.12 |
| Kidney | Mean | 96.67±7.73 | 106.36±2.98 | 97.09±2.48 |
|  | CV, % | 7.99 | 2.80 | 2.56 |
| Urine | Mean | 96.73±4.12 | 96.53±2.96 | 99.11±2.82 |
|  | CV, % | 4.27 | 3.07 | 2.71 |
| Feces | Mean | 98.21±5.64 | 104.71±2.99 | 102.76±3.46 |
|  | CV, % | 5.75 | 2.85 | 3.37 |

1. **Dilution factor (5×, 10×)**

| **Sample** | **Conc.** | **Tested conc.** | **RE** | **RSD** |
| --- | --- | --- | --- | --- |
| Plasma | 8000 | 8044.00 ± 35.13 | 0.25±1.58 | 0.44 |
|  | 16000 | 16144.00±138.94 | 5.3±0.47 | 0.86 |
| Brain | 8000 | 7580.00±132.66 | −5.25±1.66 | 1.75 |
|  | 16000 | 14740.00±241.66 | −7.88±1.51 | 1.64 |
| Heart | 8000 | 7344.00±119.18 | −8.2±1.49 | 1.62 |
|  | 16000 | 15702.00±274.77 | −1.86±1.72 | 1.75 |
| Liver | 8000 | 7772.00±122.21 | −2.85±1.53 | 1.53 |
|  | 16000 | 15638.00±155.74 | −2.26±0.97 | 0.97 |
| Spleen | 8000 | 8008.00±157.91 | 0.1±1.97 | 1.97 |
|  | 16000 | 16546.00±202.15 | 3.41±1.26 | 1.22 |
| Lung | 8000 | 8020.00±126.17 | 0.25±1.58 | 1.57 |
|  | 16000 | 16848.00±75.47 | 5.3±0.47 | 0.45 |
| Kidney | 8000 | 8268.00±59.97 | 3.35±0.75 | 0.73 |
|  | 16000 | 16546.00±137.49 | 3.41±0.86 | 0.83 |
| Urine | 8000 | 8357.00±132.57 | 4.46±1.66 | 1.59 |
|  | 16000 | 16838.00±70.26 | 5.24±0.44 | 0.42 |
| Feces | 8000 | 8107.00±125.12 | 1.34±1.56 | 1.54 |
|  | 16000 | 16614.00±158.32 | 0.04±0.01 | 0.95 |

1. **Stability**

| **Sample** | **Conc.** | **Bench top** | | **Freeze and thaw** | | **Long-term** | |
| --- | --- | --- | --- | --- | --- | --- | --- |
|  |  | **Mean** | **RSD** | **Mean** | **RSD** | **Mean** | **RSD** |
| Plasma | LQC | 26.30±0.43 | 1.63 | 25.47±0.18 | 0.71 | 25.38±0.54 | 2.12 |
|  | MQC | 824.00±41.72 | 5.06 | 777.67±5.25 | 0.68 | 880.63±23.78 | 2.70 |
|  | HQC | 1662.67±7.85 | 0.47 | 1653.00±10.03 | 0.61 | 1577.33±10.53 | 0.67 |
| Brain | LQC | 25.00±0.36 | 1.42 | 25.12±0.97 | 3.86 | 23.01±0.99 | 4.32 |
|  | MQC | 783.00±5.72 | 0.73 | 794.53±9.62 | 1.21 | 802.77±18.09 | 2.25 |
|  | HQC | 1613.33±46.43 | 2.88 | 1554.00±39.15 | 2.52 | 1525.33±23.80 | 1.56 |
| Heart | LQC | 25.79±0.46 | 1.80 | 24.93±0.97 | 3.90 | 24.83±1.71 | 6.88 |
|  | MQC | 747.30±9.67 | 1.29 | 826.37±11.65 | 1.41 | 793.00±55.55 | 7.01 |
|  | HQC | 1599.33±58.98 | 3.69 | 1475.00±29.47 | 2.00 | 1609.97±35.10 | 2.18 |
| Liver | LQC | 24.56±0.25 | 1.00 | 24.56±0.59 | 2.30 | 24.65±0.15 | 0.61 |
|  | MQC | 884.47±2.46 | 0.28 | 850.37±19.74 | 2.32 | 868.30±6.14 | 0.71 |
|  | HQC | 1620.67±5.79 | 0.36 | 1627.33±51.23 | 3.15 | 1673.33±5.79 | 0.34 |
| Spleen | LQC | 25.75±0.09 | 0.35 | 26.70±0.27 | 1.01 | 26.18±0.15 | 0.56 |
|  | MQC | 871.40±15.47 | 1.78 | 820.97±33.66 | 4.10 | 856.43±12.87 | 1.50 |
|  | HQC | 1640.00±76.44 | 4.66 | 1545.00±7.79 | 0.50 | 1564.00±38.79 | 2.48 |
| Lung | LQC | 26.90±0.11 | 0.41 | 26.23±0.55 | 2.09 | 25.51±0.43 | 1.69 |
|  | MQC | 851.10±5.89 | 0.69 | 877.70±13.52 | 1.54 | 872.63±11.64 | 1.33 |
|  | HQC | 1555.33±11.03 | 0.71 | 1615.00±31.03 | 1.92 | 1613.67±30.18 | 1.87 |
| Kidney | LQC | 26.81±0.29 | 1.09 | 26.59±0.26 | 0.97 | 25.94±0.75 | 2.87 |
|  | MQC | 801.67±47.76 | 5.96 | 818.73±55.59 | 6.79 | 810.07±50.04 | 6.18 |
|  | HQC | 1661.67±7.76 | 0.47 | 1617.33±30.35 | 1.88 | 1592.67±6.24 | 0.39 |
| Urine | LQC | 25.29±0.41 | 1.62 | 25.29±0.90 | 3.57 | 25.23±0.18 | 0.71 |
|  | MQC | 883.23±26.22 | 2.97 | 852.23±78.97 | 9.27 | 786.20±44.22 | 5.62 |
|  | HQC | 1650.67±32.43 | 1.96 | 1633.67±28.52 | 1.75 | 1655.33±25.04 | 1.51 |
| Feces | LQC | 26.28±0.02 | 0.08 | 24.52±0.94 | 3.83 | 26.59±0.29 | 1.09 |
|  | MQC | 864.07±6.78 | 0.78 | 814.30±48.03 | 5.90 | 752.37±38.10 | 5.06 |
|  | HQC | 1652.00±0.82 | 0.05 | 1660.00±31.12 | 1.87 | 1614.33±20.01 | 1.24 |
